# Cantharidin and sodium fluoride attenuate the negative inotropic effects of carbachol in the isolated human atrium

**DOI:** 10.1007/s00210-023-02747-4

**Published:** 2023-10-06

**Authors:** Rebecca Schwarz, Britt Hofmann, Ulrich Gergs, Joachim Neumann

**Affiliations:** 1 Institute for Pharmacology and Toxicology, Medical Faculty, Martin Luther University Halle-Wittenberg, Halle (Saale), Germany; 2 Department of Cardiac Surgery, Mid-German Heart Center, University Hospital Halle, Halle (Saale), Germany

**Keywords:** Cantharidin, Carbachol, Human atrium, Mouse atrium, Phosphatases, Sodium fluoride

## Abstract

Carbachol, an agonist at muscarinic receptors, exerts a negative inotropic effect in human atrium. Carbachol can activate protein phosphatases (PP1 or PP2A). We hypothesized that cantharidin or sodium fluoride, inhibitors of PP1 and PP2A, may attenuate a negative inotropic effect of carbachol. During bypass-surgery trabeculae carneae of human atrial preparations (HAP) were obtained. These trabeculae were mounted in organ baths and electrically stimulated (1 Hz). Force of contraction was measured under isometric conditions. For comparison, we studied isolated electrically stimulated left atrial preparations (LA) from mice. Cantharidin (100 µM) and sodium fluoride (3 mM) increased force of contraction in LA (*n* = 5–8, *p* < 0.05) by 113% ± 24.5% and by 100% ± 38.2% and in HAP (*n* = 13–15, *p* < 0.05) by 625% ± 169% and by 196% ± 23.5%, respectively. Carbachol (1 µM) alone exerted a rapid transient maximum negative inotropic effect in LA (*n* = 6) and HAP (*n* = 14) to 46.9% ± 3.63% and 19.4% ± 3.74%, respectively (*p* < 0.05). These negative inotropic effects were smaller in LA (*n* = 4–6) and HAP (*n* = 9–12) pretreated with 100 µM cantharidin and amounted to 58.0% ± 2.27% and 59.2% ± 6.19% or 3 mM sodium fluoride to 63.7% ± 9.84% and 46.3% ± 5.69%, (*p* < 0.05). We suggest that carbachol, at least in part, exerts a negative inotropic effect in the human atrium by stimulating the enzymatic activity of PP1 and/or PP2A.

## Introduction

**Fig. 1:**
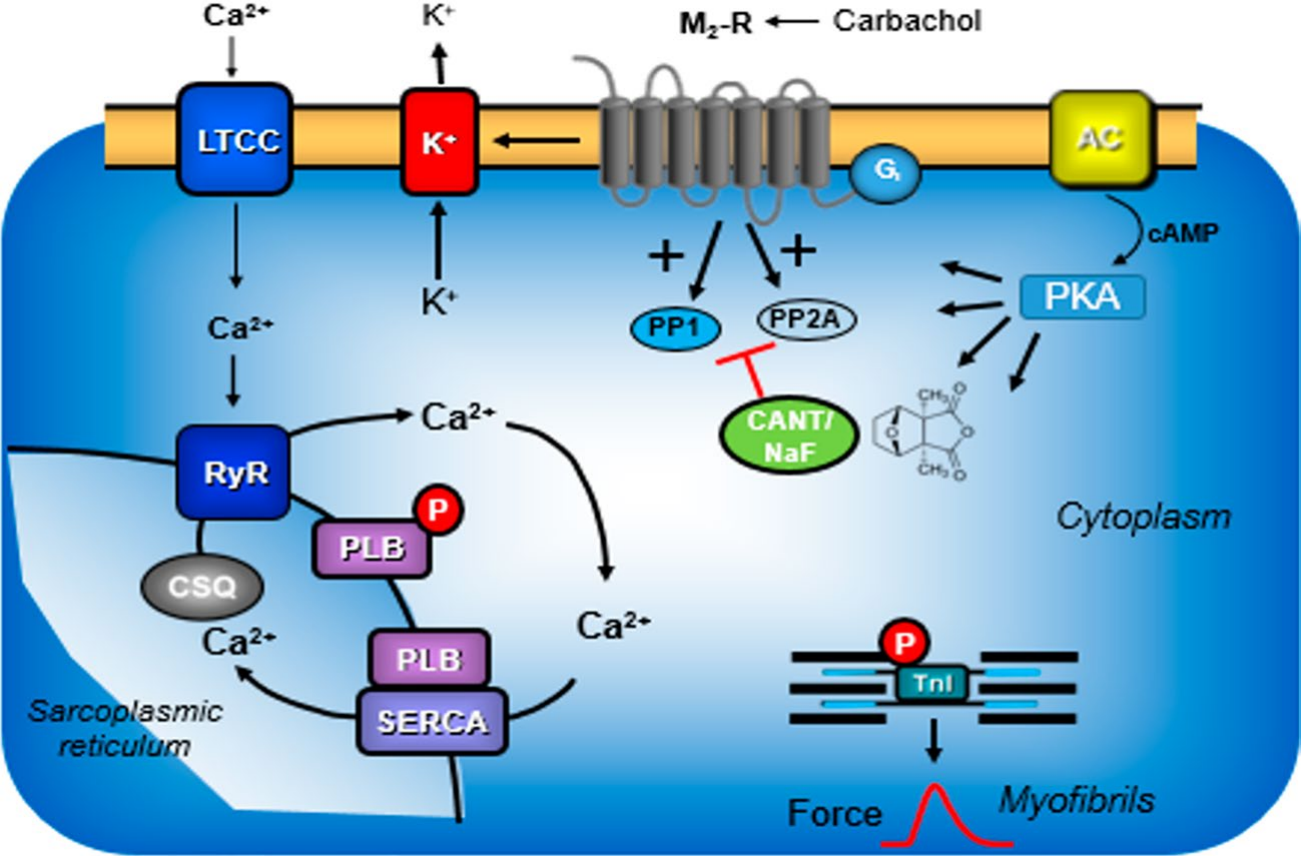
Scheme: The cardiac effects of carbachol: The Ca^2+^ for force generation is derived in part from “trigger” Ca^2+^ entering the cardiomyocyte through the L-type Ca^2+^ channel (LTCC) and in larger part by subsequent release of Ca^2+^ from the sarcoplasmic reticulum (SR) via ryanodine receptors (RYR). Cardiac relaxation is brought about by phosphorylation of phospholamban (PLB) and of inhibitory subunit of troponin (TnI). Phosphorylated PLB de-inhibits the activity of the SR—Ca^2+−^ATPase (SERCA) and Ca^2+^is more rapidly removed from the cytosol into the SR, supporting relaxation. The activities of protein phosphatases called PP1 and PP2A are inhibited by cantharidin (see structural formula as inset, a naturally occurring molecule) and by sodium fluoride. Carbachol is an agonist at cardiac M_2_-muscarinic receptors (M_2_-R). Carbachol may inhibit the enzymatic activity of adenylyl cyclases (AC) via a pertussis toxin sensitive inhibitory guanosine-triphosphate binding-protein (G_i_). Carbachol opens potassium channels (K^+^), may close the L-type Ca^2+^ channel (LTCC) and may activate PP1 and/or PP2A

Serine/threonine phosphatases (PP) are ubiquitously expressed (Herzig and Neumann 2000). Therefore, serine/threonine phosphatases are also detectable in the human heart (Lüss et al. 2000). The purposes of PP are not perfectly clear. Some say PP are continuously active, at a low turnover level and not regulated. This would serve the purpose to shut off signal transduction pathways that activate protein kinases (Fig. 1) and thereby lead to protein phosphorylation and thus activation of proteins. Another, alternative or perhaps complementary view is that PP may signal from the sarcolemma to intracellular proteins. In other words, there may exist an active coupling of sarcolemmal receptors to phosphatases. There is evidence that binding of sarcolemmal receptors to phosphatase by scaffolding complexes may occur but this matter is subject of active ongoing research (Neumann et al. 2021)

As an example, (Fig. 1) β-adrenoceptors, activate adenylate cyclase via stimulatory guanosine-triphosphate (GTP)-binding proteins and lead thereby to the formation of 3´,5´ cyclic adenosine monophosphate (cAMP). Thereafter, in the working myocardium, cAMP activates kinases (PKA) phosphorylate and thereby activate regulatory proteins. These phosphorylations are reversed by PP (Herzig and Neumann 2000, Neumann et al. 2021).

We have demonstrated in guinea pig and human preparations that cantharidin but also sodium fluoride inhibited crude but also purified catalytic subunits of PP1 and PP2A from hearts (Neumann et al. 1995a,b; Neumann and Scholz 1998). This inhibition is apparently relevant: In functional studies, we noticed that cantharidin increased force of contraction in guinea pig papillary muscles and also in human atrial and ventricular preparations via increasing the phosphorylation of regulatory proteins (Neumann et al. 1995b; Schwarz et al. 2022a, b, 2023a, b). To the best of our knowledge, no publication has yet reported on the inotropic effect of carbachol in the presence of cantharidin in human atrial preparations or mouse atrial preparations. Mouse preparations were studied here not only for comparison, but because a number of transgenic mouse models exist in which PP were genetically overexpressed or knocked out. Hence, such models might be used in subsequent studies to understand the mechanism of action of cantharidin and sodium fluoride in the atrium on a more molecular level. Sodium fluoride increased force of contraction in guinea pig ventricular preparations in a concentration-dependent fashion that peaked at 3 mM, because higher concentrations reduced force of contraction or the preparations even stopped to beat (Neumann et al. 1995a; Neumann and Scholz 1998).

Moreover, we and others have provided evidence that the ability of carbachol to reduce force of contraction at least in the presence of β-adrenoceptor stimulation in the mammalian ventricle involves not only inhibition of cAMP-production but also activation of cardiac phosphatases (Gupta et al. 1998; Herzig et al. 1995; Dhein et al. 2001).

Moreover, in the atrium as well as in the ventricle of most mammals (including humans), carbachol also can open potassium channels (Löffelholz and Pappano 1985). This shortens the action potential duration: this reduces the time in that Ca^2+^ can enter the cell via the L-type Ca^2+^channel (LTCC). As a consequence, free cytosolic concentration of Ca^2+^ in the cardiomyocyte falls. Finally, this mechanism must also contribute, at least to some extent, to the negative inotropic effect of carbachol (Löffelholz and Pappano 1985; Verkerk et al. 2022). We have shown that the inhibitory effect of carbachol on the isoprenaline-stimulated current through the LTCC is blocked by sodium fluoride and other phosphatase inhibitors like calyculin A or okadaic acid (Herzig et al. 1995). We have shown that cantharidin and sodium fluoride concentration-dependently reduced the phosphatase activity in mammalian hearts (Neumann et al. 1995a, b). Okadaic acid is more potent to inhibit cardiac phosphatase activity than cantharidin or sodium fluoride (Neumann et al. 1993). Similarly, okadaic acid starts to increase force of contraction in the guinea pig papillary muscles at lower concentrations than cantharidin or sodium fluoride, namely at 3 µM okadaic acid (Neumann et al. 1993). However, okadaic acid is a natural product and as such much more difficult to purify than cantharidin that can be synthesized with high yield in the test tube at low cost (Knapp et al. 1998). Therefore, for cost restrictions, in organ bath experiments (10 ml bath volume), we use routinely cantharidin and not okadaic acid.

The starting point for us in this context was the following reasoning. Let us assume that carbachol acts on force of contraction in mammalian myocardium, in part, by stimulation of PP1 and/or PP2A. Then, if we inhibit PP1 and/or PP2A by means of cantharidin or sodium fluoride, the negative inotropic and negative chronotropic effect of carbachol should be attenuated. We have shown an interaction of cantharidin and carbachol before by measuring protein phosphorylation in quiescent guinea pig ventricular cardiomyocytes and contraction in ventricular preparations from guinea pig (Neumann et al. 1995b). Here, we hypothesize that such an interaction also exists in atrial tissue being aware that functional differences in the signal transduction between the atrium and the ventricle of mammalian hearts exist.

Similar studies in the isolated mammalian atrium are hitherto lacking. Nevertheless, carbachol or acetylcholine can reduce force of contraction in atrial preparations. More specifically, there is a biphasic effect of carbachol in the mouse atrium (e.g. Boknik et al. 2009) as well as the human atrium (Du et al. 1994, 1995). Initially, carbachol induces a strong negative inotropic effect in atrial preparations from mouse, guinea pig and humans. This is rapidly followed by a positive inotropic effect. Using mouse gene deletion studies and receptor specific antagonists, the following is clear: all five muscarinic receptors are present in the human heart. M_2_-muscarinic receptors are the most abundant in the heart. M_2_-muscarinic receptors mediate the negative inotropic effect (Dhein et al. 2001).

To sum it up, we tested the following main hypotheses: Firstly, cantharidin may attenuate the negative inotropic effect of carbachol in the isolated mouse and human atrium. Secondly, sodium fluoride may attenuate the negative inotropic effect of carbachol in the isolated human and mouse atrium. Progress reports of this work have been published in abstract form (Schwarz et al. 2022a, b, 2023a).

## Materials and methods

### Contractile studies in mice

In brief, left atrial preparations from the (wild type) mice were isolated and mounted in organ baths as previously described (Gergs et al. 2013; Neumann et al. 2003). The bathing solution of the organ baths contained 119.8 mM NaCI, 5.4 mM KCI, 1.8 mM CaCl_2_, 1.05 mM MgCl_2_, 0.42 mM NaH_2_PO_4_, 22.6 mM NaHCO_3_, 0.05 mM Na_2_EDTA, 0.28 mM ascorbic acid and 5.05 mM glucose. The solution was continuously gassed with 95% O_2_ and 5% CO_2_ and maintained at 37 °C and pH 7.4 (Neumann et al. 2003).

The drug application was as follows. After equilibration was reached, 100 µM cantharidin or 3 mM sodium fluoride were added to left atrial preparations until the maximum positive inotropic effect was reached. Then carbachol was cumulatively applied to the preparations. As depicted in the legends, in some experiments the next higher concentrations of carbachol were applied only when the negative inotropic had reached its maximum. In other experiments, the next carbachol concentration was added when the positive inotropic effect of carbachol had reached a plateau.

### Contractile studies on human preparations

The contractile studies on human preparations were done using the same setup and buffer as used in the mouse studies (see Sect. 2.1). The samples were obtained from five female and 13 male patients aged 47–86 years. Drug therapy included metoprolol, furosemide, apixaban and acetyl salicylic acid. Our methods used for atrial contraction studies in human samples have been previously published and were not altered in this study (Gergs et al. 2017, 2018; Boknik et al. 2019). Written informed consent was obtained for the use of right atrial tissues from patients undergoing cardiac surgery.

### Western blotting

The homogenization of the samples, protein measurements, electrophoresis, primary and secondary antibody incubation and quantification were performed following our previously with slight modifications (Gergs et al. 2009, 2019; Boknik et al. 2018). Electrophoresis was performed in Novex™ 4–20% “Tris–Glycine Plus Midi Protein Gels” (Invitrogen, Thermo Fisher Scientific, Waltham, Massachusetts, USA). The run was performed at 4 °C for approximately 1.5 h at 120 V in the “NuPAGE MES SDS Running Buffer” (Thermo Fisher Scientific, Waltham, Massachusetts, USA) using an XCell4 SureLock™ Midi-Cell chamber (Life Technologies by Thermo Fisher Scientific, Waltham, Massachusetts, USA). Protein transfer into membranes (Amersham™ Protran, GE Healthcare, Chicago, Illinois, USA) was performed at 195 mA for 20 h at 4 °C. Membrane blocking for 1 h at room temperature was followed by overnight incubation at 4 °C with the primary antibody for serine 16-phosphorylated phospholamban (1:5000; catalog number: A010-12AP; PLB Ser16; Badrilla, Leeds, UK), phospho-troponin I (1:5000; catalog number: 4004; Ser23/24; cell signaling technology, Leiden, the Netherlands). While calsequestrin antibody was used as a loading control (1:20.000; product number: ab3516; abcam, Cambridge, UK). Visualization of the signals was performed by using a chemiluminescence substrate (Immobilon™ Western, Millipore, Merck; Darmstadt, Germany) and a digital imaging system (Amersham ImageQuant 800; Cytiva Europe GmbH, Freiburg im Breisgau, Germany).

### Data analysis

Data shown are means ± standard error of the mean. Statistical significance was estimated using the analysis of variance followed by Bonferroni’s t-test. A p-value < 0.05 was considered to be significant.

### Drugs and materials

The drugs isoprenaline-hydrochloride, cantharidin (CANT, 100 mM dissolved in dimethylsulfoxide (DMSO)), sodium fluoride and carbachol (CAR) were purchased from Sigma-Aldrich (Germany). All other chemicals were of the highest purity grade commercially available. Deionized water was used throughout the experiments. Stock solutions were prepared fresh daily.

## Results

### Cantharidin

#### Mouse left atrium

**Fig. 2:**
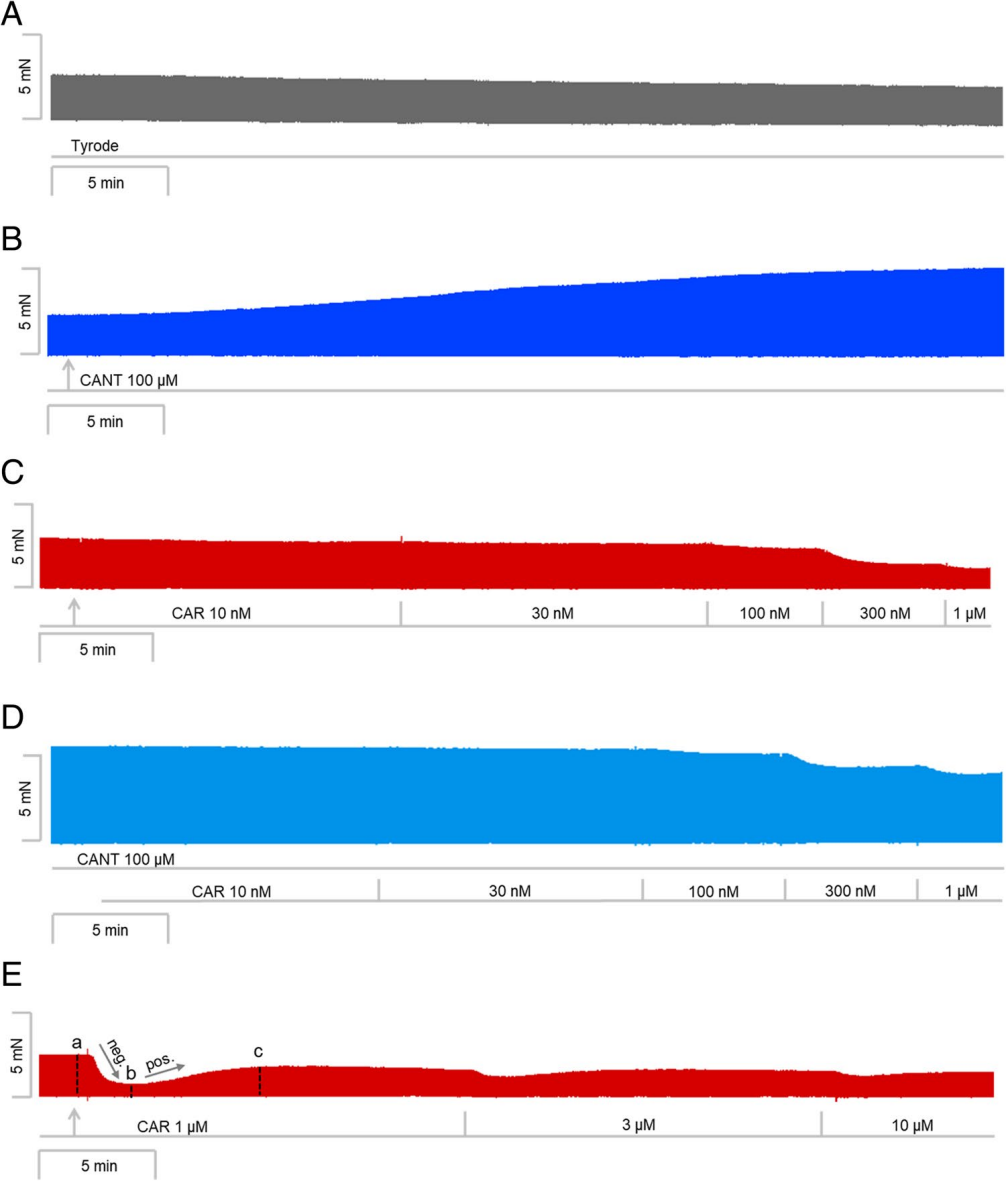

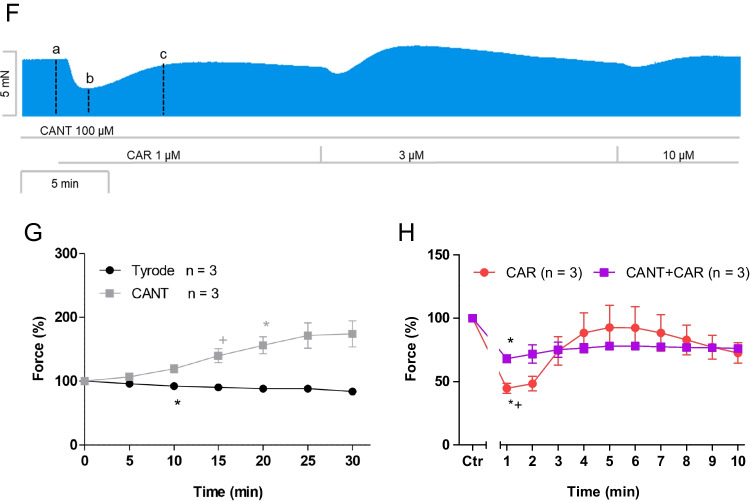
Cantharidin attenuates the negative inotropic effect of carbachol on force of contraction in mouse left atrium. Original recording of the buffer, Tyrode’s solution, alone (**A**), in the presence of 100 µM cantharidin alone (**B**), after application of carbachol (10, 30, 100, 300 nM, 1 µM) alone (**C**) or with cantharidin (100 µM) first applied (**D**). Concentrations of carbachol applied before negative inotropic effect was reversed by subsequent positive inotropic effect to carbachol and after application of carbachol (1, 3, 10 µM) alone (**E**), or if applying first cantharidin (100 µM) and, when plateau was reached, thereafter carbachol for longer periods of time such that the positive inotropic effects of carbachol that followed the negative inotropic effect of carbachol reached plateau (1, 3, 10 µM, **F**). The ordinates give force of contraction in milli Newton (mN) over time in minutes (min, abscissae) in electrically stimulated wild type mouse left atrium. The ratio of force at label ***b*** divided by force at label ***a*** marks the negative inotropic effect of carbachol. **G** Summarized data indicating the positive inotropic effect of 100 µM cantharidin over time. Ordinate and abscissa give force of contraction in % of pre-drug value or time in minutes (min), respectively. * and  + first significant difference versus zero time point (Ctr) or Tyrode´s solution, respectively. Number of experiments *n* = 3. **H** Summarized data indicating the negative and positive inotropic effect of carbachol (1 µM) in presence or absence of cantharidin (100 µM) over time. Ordinate and abscissa give force of contraction in % of pre-drug value or time in minutes (min), respectively. * and  + first significant difference versus zero time point (ctr) or carbachol in presence of cantharidin, respectively. Number of experiments *n* = 3

We have published recently that cumulatively applied cantharidin exerted a concentration- and time-dependent positive inotropic effect in left atrial preparations from mice (Schwarz et al. 2023b). Here, we utilized a single concentration of cantharidin (100 µM), the highest concentration we studied before (Schwarz et al. 2023b). Cantharidin (100 µM) exerted a time-dependent positive inotropic effect (Fig. 2B) compared to a time control experiment (Fig. 2A). The time course of the positive inotropic effect of cantharidin was quantified in Fig. 2G. The positive inotropic effect gained significance after an incubation time of 15 min.

**Fig. 3:**
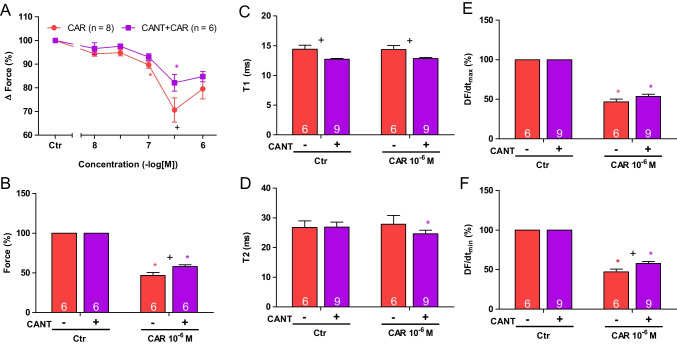
Cantharidin attenuates the negative inotropic effect of carbachol on contractile parameters in mouse left atrium. **A** Negative inotropic effect of carbachol (10, 30, 100, 300 nM, 1 µM) alone or after previous application of cantharidin (100 µM) in isolated electrically driven mouse left atrial preparations. In the pipetting of the concentration response curves, the subsequent concentration of carbachol was applied just before the negative inotropic effect was reversed to a positive inotropic effect. Ordinate and abscissa give force of contraction in % of pre-drug value or applied concentration of carbachol, respectively. * and  + first significant difference versus zero time point (Ctr) or carbachol in presence of cantharidin, respectively. Numbers in brackets indicate the number of experiments. **B** Bar diagram of the negative inotropic effect of carbachol (1 µM) alone or in the additional presence of cantharidin (100 µM) in isolated electrically driven mouse left atrial preparations. The effect of 100 µM cantharidin or time matched control values on force of contraction were set as 100 percent (left two columns). Negative inotropic effects of carbachol in the absence (-) or in the presence (+) of 100 µM cantharidin are plotted. *p < 0.05 vs. zero time point (Ctr). +  *p* < 0.05 vs. without cantharidin. Ordinate: Force of contraction % of the value after or before drug addition. Numbers in bars indicate number of experiments. **C** Bar diagram of the effect of carbachol alone (1 µM) or in the additional presence of cantharidin (100 µM) on time to peak tension in isolated electrically driven mouse left atrial preparations. The effects of 100 µM cantharidin alone or time matched control values for time to peak tension are shown as control (Ctr) (left two columns). Next is displayed the effect of carbachol in the absence (-) or in the presence (+) of 100 µM cantharidin on time to peak tension. +  *p* < 0.05 vs. without cantharidin. Ordinate: time to peak tension in milliseconds (ms). Numbers in bars indicate the number of experiments. **D** Bar diagram of the effect of carbachol (1 µM) alone or in the additional presence of cantharidin (100 µM) on time of relaxation in isolated electrically driven mouse left atrial preparations. The effect of 100 µM cantharidin or time matched control values for time of relaxation are shown (left two columns). Next is displayed the effect of carbachol in the absence (-) or in the presence (+) of 100 µM cantharidin on time of relaxation. * *p* < 0.05 vs. zero time point (Ctr). Ordinate: time of relaxation in milliseconds (ms). Numbers in bars indicate number of experiments. **E** Bar diagram of the effect of carbachol (1 µM) alone or in the additional presence of cantharidin (100 µM) on rate of tension development in isolated electrically driven mouse left atrial preparations. The effect of 100 µM cantharidin or time matched control values for rate of tension development were set as 100 percent (left two columns). Effects of carbachol in the absence (-) or in the presence (+) of 100 µM cantharidin on rate of tension development are plotted. *p < 0.05 vs. zero time point (Ctr). Ordinate: rate of tension development in %. Numbers in bars indicate number of experiments. **F** Bar diagram of the effect of carbachol (1 µM) alone or in the additional presence of cantharidin (100 µM) on rate of tension relaxation in isolated electrically driven mouse left atrial preparations. The effect of 100 µM cantharidin or time matched control values on rate of tension relaxation were set as 100 percent (left two columns). Effects of carbachol in the absence (-) or in the presence (+) of 100 µM cantharidin on rate of tension relaxation are plotted. *p < 0.05 vs. zero time point (Ctr). + *p* < 0.05 vs. without cantharidin. Ordinate: rate of tension relaxation in %. Numbers in bars indicate number of experiments

Next, two different modes of application of carbachol were studied in order to discern in detail the contractile effects of carbachol. In the first set of experiments, we only examined the negative inotropic effect of carbachol. To that end, carbachol (10 nM—1 µM) was cumulatively applied, and the next higher concentration was applied before a positive inotropic effect of carbachol could develop. Experiments with carbachol alone (Fig. 2C) or after pre-incubation with 100 µM cantharidin for 30 min (Fig. 2D) were performed. It turned out that cantharidin can attenuate the negative inotropic effects of carbachol (Fig. 3A).

In the second set of experiments, we wanted to obtain a steady state for the complete response to carbachol at each concentration. In other words, we waited until the transient negative inotropic effect of carbachol was followed by a stable positive inotropic effect before the next higher concentration was applied (Fig. 2E). The data are summarized in Table 1. It turned out again that cantharidin can attenuate the negative inotropic effects of carbachol (Table 1). In detail, 1 µM carbachol elicited a pronounced negative inotropic effect followed by a positive inotropic effect and then a plateau was reached (Fig. 2E). At 3 µM and 10 µM carbachol, cumulatively applied, the negative and positive inotropic effects of carbachol were smaller than at 1 µM carbachol (Fig. 2E). In separate experiments, first cantharidin was applied for 30 min as in Fig. 2B and then carbachol was applied as in Fig. 2F. In the presence of cantharidin, the negative inotropic effect of carbachol at 1 µM is attenuated (Fig. 2F). These data are summarized in Fig. 3B. Likewise, under these conditions, the reductions of the rate of tension development and time of relaxation by 1 µM carbachol were attenuated in the presence of cantharidin (Fig. 3D, E and Table 1). Under these conditions, time to peak tension was shortened by cantharidin (Fig. 3C) and was unchanged by carbachol in the presence or absence of cantharidin (Fig. 3C and Table 1). Cantharidin (100 µM) alone did not shorten time of relaxation by its own (Fig. 3D), but additionally applied carbachol shortened time of relaxation (Fig. 3D).

**Table 1:** Summarized data of force of contraction (Force, mN), time to peak tension (t1, ms) and time to relaxation (t2, ms), rate of tension development (dF/dtmax, mN/s) and rate of tension relaxation (dF/dtmin, mN/s) of mouse LA and HAP for carbachol alone (CAR) and in presence of 100 µM cantharidin (CANT + CAR) or 3 mM sodium fluoride (NaF + CAR). Ctr is equivalent to tyrode solution for carbachol alone, to 100 µM cantharidin for carbachol in presence of cantharidin and to 3 mM sodium fluoride for carbachol in presence of sodium fluoride. * *p* < 0.05 vs. Ctr, +  *p* < 0.05 carbachol versus cantharidin plus carbachol, # *p* < 0.05 carbachol versus carbachol plus sodium fluoride

Mouse		Ctr	CAR 1 µM neg	CAR 3 µM neg	CAR 10 µM neg
CAR (*n* = 6)	Force (mN)	2.27 ± 0.31	1.09 ± 0.20*	1.69 ± 0.27	1.80 ± 0.32
	t1 (ms)	14.4 ± 0.64	14.4 ± 0.69	14.1 ± 0.62	14.2 ± 0.62
	t2 (ms)	27.2 ± 2.62	28.5 ± 3.39	27.9 ± 3.18	27.8 ± 3.34
	dF/dtmax (m N/s)	146 ± 24.0	69.7 ± 15.2*	110 ± 19.9	117 ± 24.6
	dF/dtmin (m N/s)	-90.4 ± 13.4	-43.4 ± 8.51*	-67.3 ± 10.3	-71.4 ± 12.6
CANT + CAR (*n* = 6)	Force (mN)	3.84 ± 0.97	2.21 ± 0.56* +	2.31 ± 0.54*	2.53 ± 0.52
	t1 (ms)	12.7 ± 0.14	12.9 ± 0.18	13.0 ± 0.16	13.2 ± 0.16
	t2 (ms)	26.9 ± 1.70	24.7 ± 1.19*	26.7 ± 1.48	27.2 ± 1.63
	dF/dtmax (m N/s)	297 ± 55.3 +	159 ± 29.9* +	201 ± 47.0*	214 ± 46.4
	dF/dtmin (m N/s)	-156 ± 25.2 +	-90.9 ± 15.4* +	-106 ± 20.9*	-113 ± 20.2
NaF + CAR (*n* = 4)	Force (mN)	4.01 ± 0.79#	2.54 ± 0.72#	2.33 ± 0.53*	2.14 ± 0.40*
	t1 (ms)	15.6 ± 0.56	17.2 ± 0.71	17.2 ± 0.71	17.4 ± 0.76
	t2 (ms)	43.0 ± 1.46	44.3 ± 1.81#	42.4 ± 1.47#	41.3 ± 1.60#
	dF/dtmax (m N/s)	266 ± 16.9#	146 ± 37.3*#	145 ± 20.5*	130 ± 13.4*
	dF/dtmin (m N/s)	-117 ± 9.70	-67.2 ± 17.2*	-68.2 ± 8.80*	-63.4 ± 6.48*
Human		Ctr	CAR 1 µM neg	CAR 3 µM neg	CAR 10 µM neg
CAR (*n* = 14)	Force (mN)	4.80 ± 0.92	0.92 ± 0.26*	2.43 ± 0.67*	2.67 ± 0.71*
	t1 (ms)	55.8 ± 1.66	39.2 ± 3.02*	48.2 ± 3.79	48.6 ± 4.00
	t2 (ms)	124 ± 5.23	103 ± 9.99*	119 ± 6.34	116 ± 5.11
	dF/dtmax (m N/s)	82.4 ± 15.4	19.5 ± 4.02*	45.7 ± 11.5*	50.9 ± 12.4*
	dF/dtmin (m N/s)	-45.3 ± 7.19	-10.8 ± 2.28*	-22.6 ± 4.92*	-25.0 ± 5.29
CANT + CAR (*n* = 9)	Force (mN)	4.25 ± 0.65	2.67 ± 0.67* +	2.18 ± 0.57*	2.12 ± 0.51*
	t1 (ms)	45.3 ± 2.16 +	41.0 ± 2.09	38.7 ± 2.41	39.9 ± 1.94
	t2 (ms)	90.5 ± 4.49 +	81.2 ± 7.43 +	78.9 ± 8.79 +	81.8 ± 7.69 +
	dF/dtmax (m N/s)	90.0 ± 13.3	57.2 ± 11.2* +	47.4 ± 9.33*	46.1 ± 8.62*
	dF/dtmin (m N/s)	-51.3 ± 7.53	-36,3 ± 7.19* +	-31.2 ± 6.54*	-30.6 ± 6.46*
NaF + CAR (*n* = 12)	Force (mN)	3.79 ± 1.07	1.74 ± 0.58*	1.99 ± 0.68*	1.90 ± 0.66*
	t1 (ms)	81.9 ± 8.02#	83.0 ± 8.97#	80.5 ± 8.98#	79.9 ± 9.17#
	t2 (ms)	152 ± 13.5#	164 ± 16.8#	158 ± 15.9#	161 ± 18.1#
	dF/dtmax (m N/s)	54.6 ± 17.9	28.0 ± 10.3*	30.8 ± 11.1*	30.2 ± 11.3*
	dF/dtmin (m N/s)	-36.1 ± 12.8	-19.7 ± 8.24*	-20.8 ± 8.03	-20.3 ± 8.05*

In a third set of experiments, only 1 µM carbachol was given, and its effect was quantified for 10 min. For comparison, in separate experiments, first 100 µM cantharidin was given for 30 min and then, in addition, carbachol (1 µM) was applied for 10 min and force monitored. The data are presented in Fig. 2H. Here, one minute after application, the negative inotropic effect of carbachol is smaller in the presence than in the absence of cantharidin (Fig. 2H).

### Human right atrial preparations

**Fig. 4:**
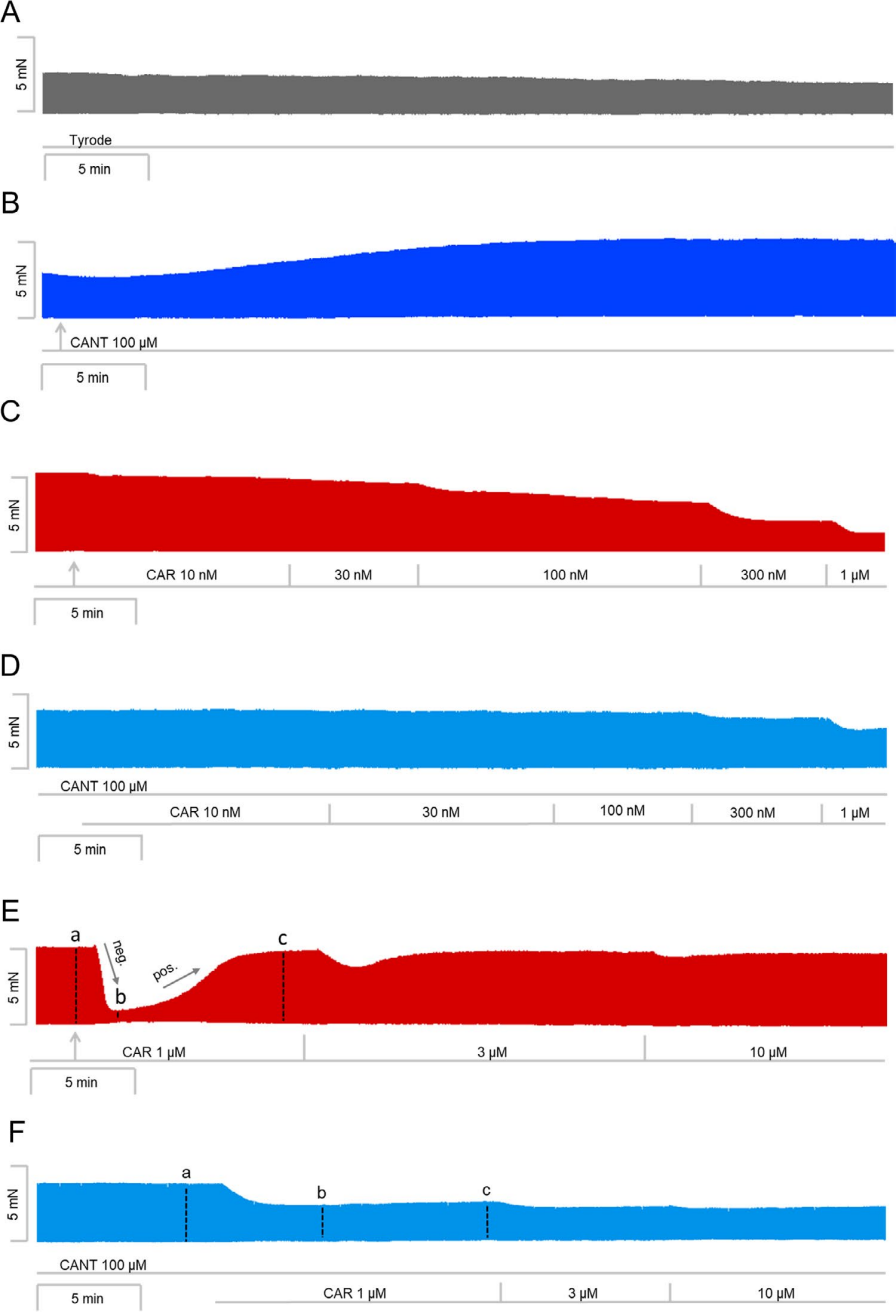

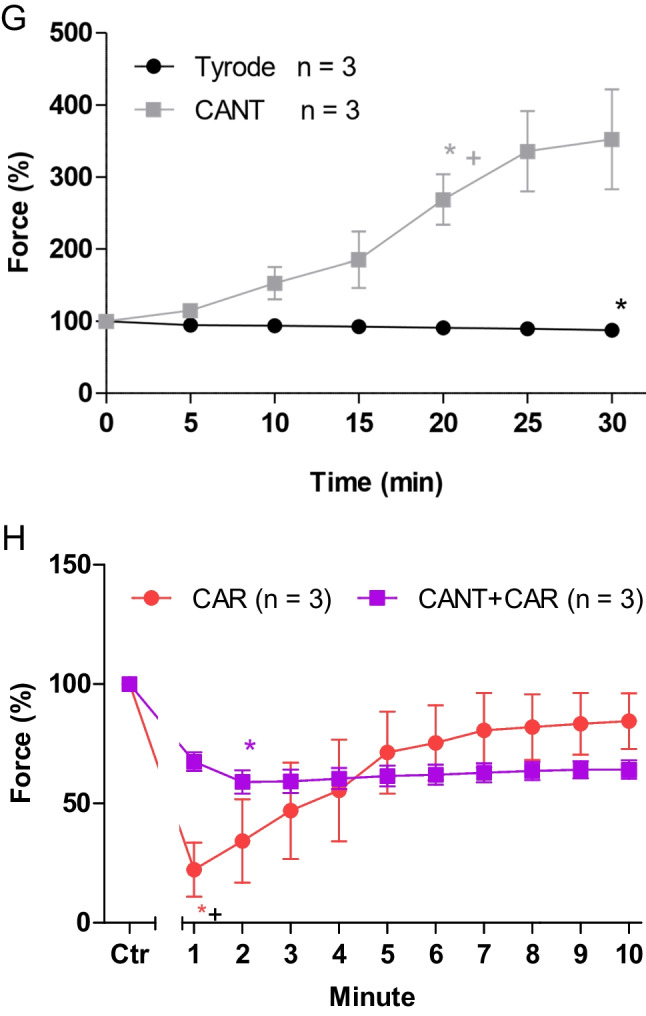
Cantharidin attenuates the negative inotropic effect of carbachol on force of contraction in human right atrium**.** Original recordings of the buffer, Tyrode’s solution, alone (**A**), in the presence of 100 µM cantharidin alone (**B**), after application of carbachol (10, 30, 100, 300 nM, 1 µM) alone (**C**) or with cantharidin (100 µM) first applied (**D**) followed by a carbachol-induced concentration response curve. Concentrations of carbachol were applied before the negative inotropic effect was reversed and after application of carbachol (1, 3, 10 µM) alone (**E**), or if applying first cantharidin (100 µM) and, when plateau was reached, thereafter carbachol (1, 3, 10 µM, **F**). The ordinates give force of contraction in milli Newton (mN, ordinates) over time in minutes (min, abscissae) in electrically stimulated mouse left atrium. The ratio of force at label ***b*** divided by force at label ***a*** marks the negative inotropic effect of carbachol. **G** Summarized data indicating the positive inotropic effect of 100 µM cantharidin over time. Data are given in mean values in percentage ± SEM. * and  + first significant difference versus zero time point or Tyrode´s solution, respectively. Number of experiments *n* = 3. **H** Summarized data indicating the negative and positive inotropic effect of carbachol (1 µM) in presence or absence of cantharidin (100 µM) in a time-dependent manner. Ordinate and abscissa give force of contraction in % of pre-drug value or time in minutes (min), respectively. * and  + first significant difference versus zero time point (Ctr) or carbachol in presence of cantharidin, respectively. Number of experiments *n* = 3

The same protocol was used in human atrial preparations as in mice. Cantharidin (100 µM) exerted a time-dependent positive inotropic effect (Fig. 4B) compared to a time control experiment (Fig. 4A). The time course of the positive inotropic effect of cantharidin was quantified in Fig. 4G. The positive inotropic effect gained significance after an incubation time of 20 min. We chose this concentration of cantharidin in the human atrium, because we had published before concentration response curves for the positive inotropic effect of cantharidin in the human ventricle but also in muscle strips from the human atrium and had thus established that this concentration of cantharidin led to stable increases of contraction without leading to contracture that we observed sometimes at 300 µM cantharidin (Schwarz et al. 2023a, b). Moreover, we wanted to test the same concentration of cantharidin in mouse and human atria, in order to facilitate a direct comparison of the data.

**Fig. 5:**
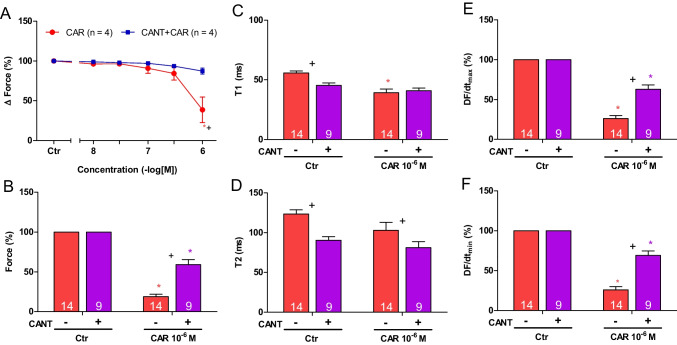

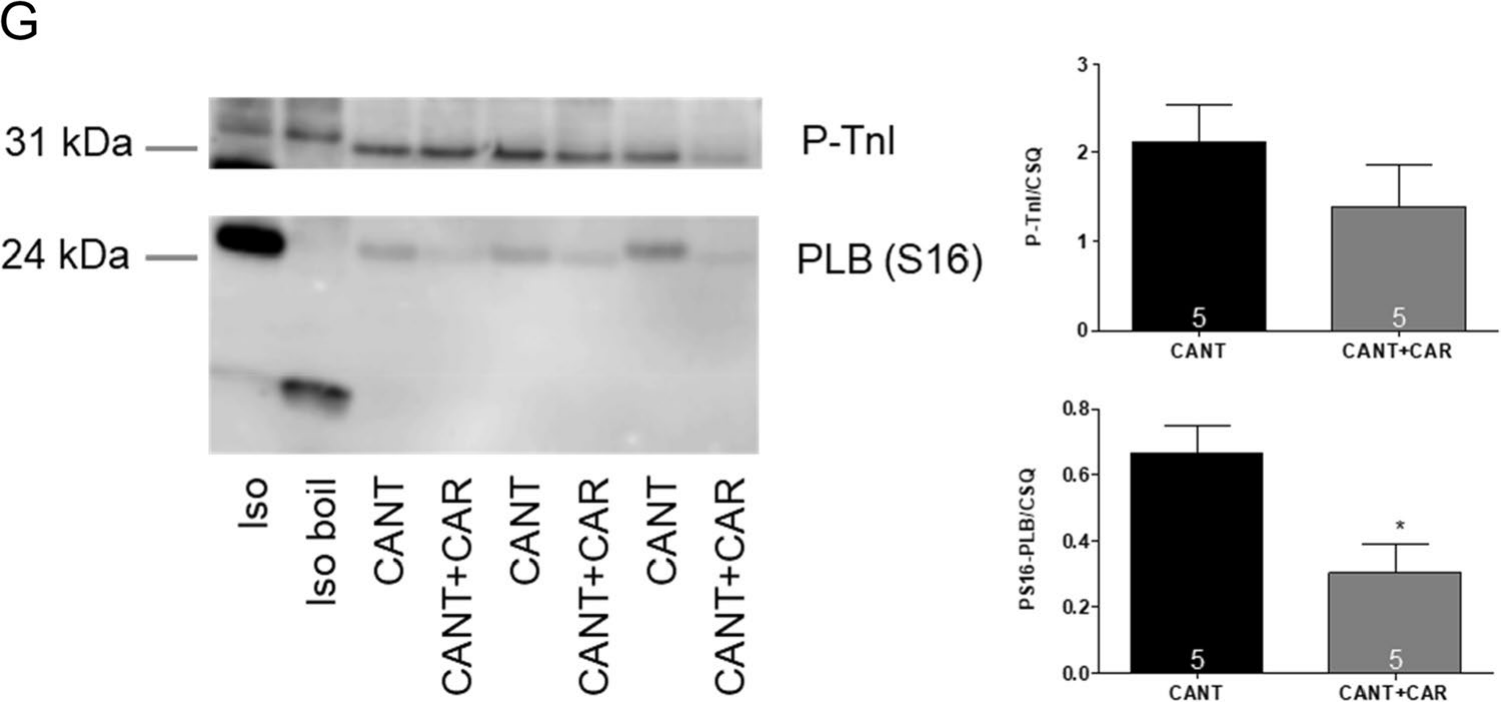
Cantharidin attenuates the contractile effects of carbachol on force of contraction in human right atrium. **A** Line diagram of the negative inotropic effect of carbachol (10, 30, 100, 300 nM, 1 µM) alone or with first applied cantharidin (100 µM) in isolated electrically driven human right atrial preparations. Subsequent concentrations of carbachol were applied to the organ bath before the negative inotropic effect was reversed. Ordinate and abscissa give force of contraction in % of pre-drug value or applied concentration of carbachol, respectively. * and  + first significant difference versus zero time point (Ctr) or carbachol in presence of cantharidin, respectively. Numbers (n) indicate number of experiments. **B** Bar diagram of the negative inotropic effect of carbachol (1 µM) alone or in the additional presence of cantharidin (100 µM) in isolated electrically driven human right atrial preparations. The effect of 100 µM cantharidin or time matched control values on force of contraction were set as 100 percent (left two columns). Negative inotropic effects of carbachol in the absence (-) or in the presence (+) of 100 µM cantharidin are plotted. * *p* < 0.05 vs. zero time point (Ctr). + *p* < 0.05 vs. without cantharidin. Ordinate: Force of contraction in %. Numbers in bars indicate number of experiments. **C** Bar diagram of the effect of carbachol (1 µM) alone or in the additional presence of cantharidin (100 µM) on time to peak tension in isolated electrically driven human right atrial preparations. The effect of 100 µM cantharidin or time matched control values for time to peak tension are shown (left two columns). Next is displayed the effect of carbachol in the absence (-) or in the presence (+) of 100 µM cantharidin on time to peak tension. * *p* < 0.05 vs. zero time point (Ctr). + *p* < 0.05 vs. without cantharidin. Ordinate: time to peak tension in milliseconds (ms). Numbers in bars indicate number of experiments. **D** Bar diagram of the effect of carbachol (1 µM) alone or in the additional presence of cantharidin (100 µM) on time of relaxation in isolated electrically driven human right atrial preparations. The effect of 100 µM cantharidin or time matched control values for time of relaxation are shown (left two columns). Next is displayed the effect of carbachol in the absence (-) or in the presence (+) of 100 µM cantharidin on time of relaxation. + *p* < 0.05 vs. without cantharidin. Ordinate: time of relaxation in milliseconds (ms). Numbers in bars indicate number of experiments. **E** Bar diagram of the effect of carbachol (1 µM) alone or in the additional presence of cantharidin (100 µM) on rate of tension development in isolated electrically driven human right atrial preparations. The effect of 100 µM cantharidin or time matched control values on rate of tension development were set as 100 percent (left two columns). Effects of carbachol in the absence (-) or in the presence (+) of 100 µM cantharidin on rate of tension development are plotted. * *p* < 0.05 vs. zero time point (Ctr). + *p* < 0.05 vs. without cantharidin. Ordinate: rate of tension development in %. Numbers in bars indicate number of experiments. **F** Bar diagram of the effect of carbachol (1 µM) alone or in the additional presence of cantharidin (100 µM) on rate of tension relaxation in isolated electrically driven human right atrial preparations. The effect of 100 µM cantharidin or time matched control values on rate of tension relaxation were set as 100 percent (left two columns). Effect of carbachol in the absence (-) or in the presence (+) of 100 µM cantharidin on rate of tension relaxation are plotted. * *p* < 0.05 vs. zero time point (Ctr). + *p* < 0.05 vs. without cantharidin. Ordinate: rate of tension relaxation in %. Numbers in bars indicate number of experiments. **G** Original Western blot of phosphorylated phospholamban at serine 16 and the phosphorylated inhibitory subunit of troponin (TnI) in contracting human atrium treated with cantharidin (100 µM) or cantharidin (100 µM) plus carbachol (1 µM). A sample treated with isoprenaline (1 µM) unboiled and boiled (95 °C for 10 min) was included as a positive control. Mobility shift in the boiled sample confirmed that we really detected phospholamban. Horizontal arrows indicate the apparent molecular weight of phospholamban phosphorylated at serine 16 (PS16-PLB) or phosphorylated inhibitory subunit of troponin (P-TnI). Bar diagrams of relative expression of phospholamban at serine 16 and phosphorylated inhibitory subunit of troponin (P-TnI) in human atrium treated with cantharidin (100 µM) or additionally treated with carbachol (1 µM) are given. Expression was normalized to calsequestrin (CSQ). * *p* < 0.05 vs. cantharidin alone. Numbers in bars indicate number of samples

Again, different modes of application of carbachol were studied in order to discern in detail the contractile effects of carbachol. In the first set of experiments, carbachol (10 nM—1 µM) was cumulatively applied with the next higher concentration before a positive inotropic effect of carbachol could develop. Again, experiments with carbachol alone (Fig. 4C) and with first applied 100 µM cantharidin (Fig. 4D) were done. It turned out that also in the human atrium, cantharidin can attenuate the negative inotropic effects of carbachol (Fig. 5A).

In the second set of experiments, we wanted to obtain a steady state for the complete response to carbachol at each concentration, as described for mouse left atria. The procedure was the same as described above for the mouse preparations (Fig. 4E). The data are summarized in Table 1. It turned out again that cantharidin can attenuated the negative inotropic effects of carbachol (Table 1). The course of carbachol effects was the same as noted for mouse left atrial preparations: 1 µM carbachol elicited a pronounced negative inotropic effect followed by a positive inotropic effect and then a plateau was reached (Fig. 4E). At 3 µM carbachol and 10 µM carbachol, cumulatively applied, the negative and positive inotropic effects of carbachol are smaller than at 1 µM carbachol (Fig. 4E).

In separate experiments, initially cantharidin was applied for 30 min as in Fig. 4B and then carbachol was applied as in Fig. 4E. In the presence of cantharidin, the negative inotropic effect of carbachol at 1 µM was attenuated (Fig. 4F). Interestingly, in human atrial preparations, also the positive inotropic effect of carbachol was attenuated by cantharidin. The data are summarized in Fig. 5B. Likewise, under these conditions, the reductions by 1 µM carbachol of the rate of tension development and time of relaxation were attenuated in the presence of cantharidin (Fig. 5D, E and Table 1). Moreover, time to peak tension was shortened by cantharidin (Fig. 5C). Whereas time to peak tension was shortened by carbachol in the absence of cantharidin, carbachol did not shorten time to peak tension in the presence of cantharidin (Fig. 5C and Table 1). Cantharidin (100 µM) alone shortened time of relaxation (Fig. 5D), but additionally applied carbachol did not further shorten time of relaxation (Fig. 5D). In human atrial preparations, the carbachol-mediated reductions of the rate of tension development (Fig. 5E) and the rate of relaxation (Fig. 5F) were attenuated by cantharidin.

Cantharidin is known to increase the phosphorylation state of phospholamban and troponin I (Schwarz et al. 2023a, b). Additionally applied carbachol reduced the phosphorylation state of phospholamban at serine 16 and tendentially of troponin I (Fig. 5G).

### Sodium fluoride

In the following part of the study, cantharidin was replaced by sodium fluoride as protein phosphatase inhibitor. Therefore, the same experiments with mouse and human atrial preparations were performed as described above, but with sodium fluoride instead of cantharidin.

### Mouse left atrium

**Fig. 6:**
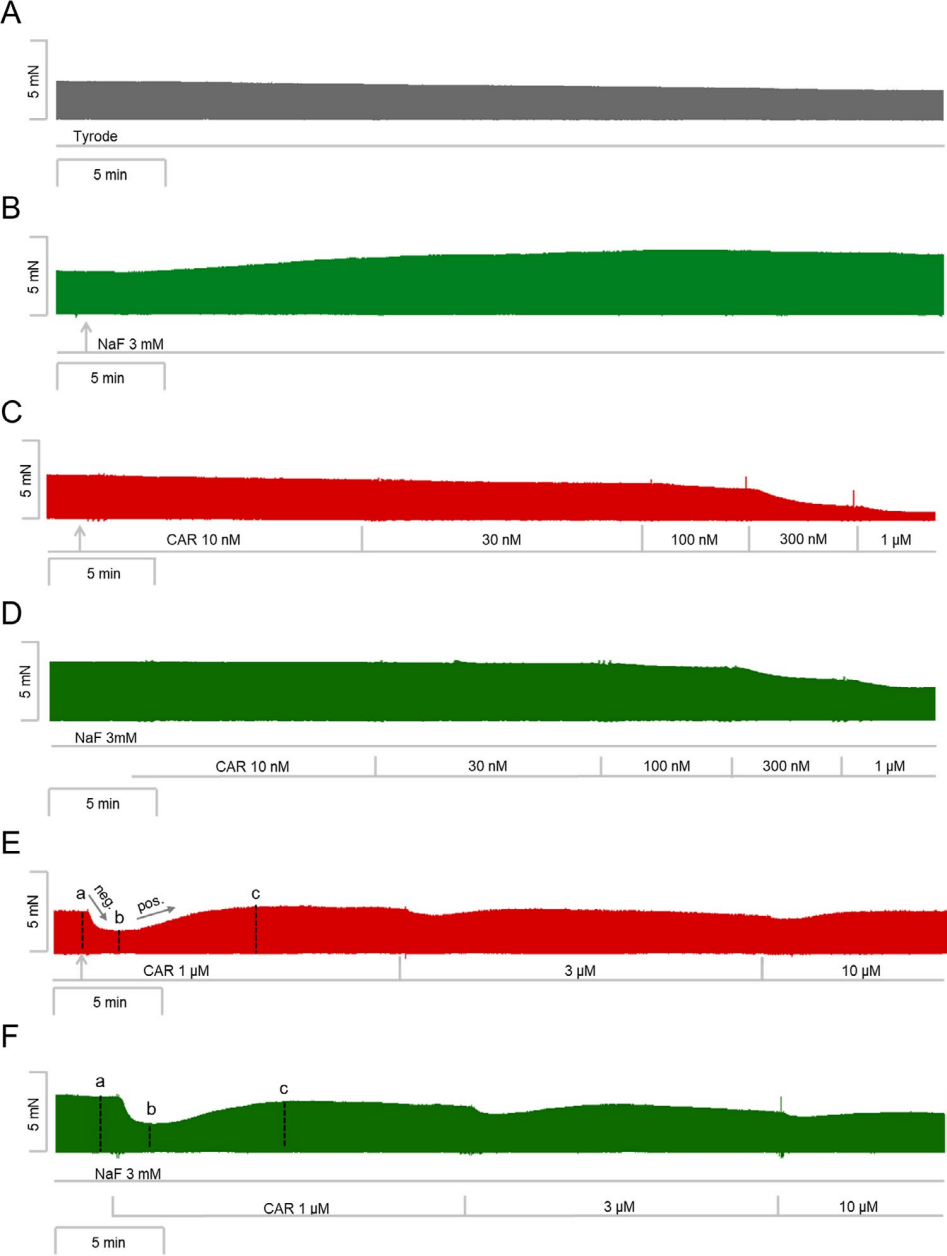

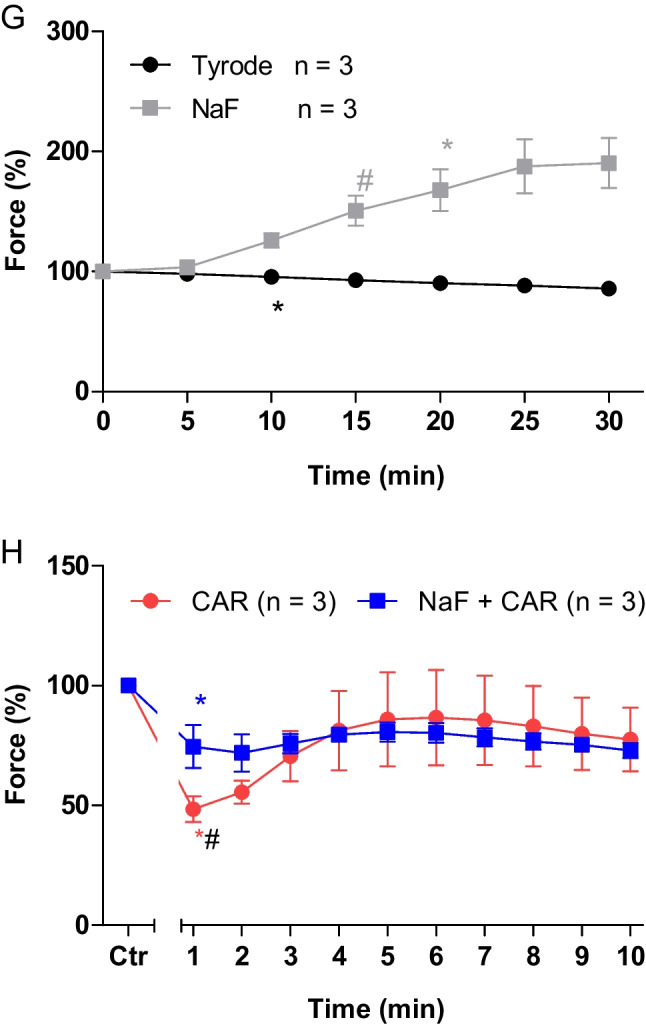
Sodium fluoride attenuates the negative inotropic effect of carbachol on force of contraction in isolated electrically driven mouse left atrial preparations. Original recording of the buffer, Tyrode’s solution, alone (**A**), in the presence of 3 mM sodium fluoride alone (**B**), after application of carbachol (10, 30, 100, 300 nM, 1 µM) alone (**C**) or with sodium fluoride (3 mM) first applied (**D**). Concentrations of carbachol were applied before negative inotropic effect was reversed at longer incubation times or after application of carbachol (1, 3, 10 µM) alone for a prolonged time such that a positive inotropic effect could appear, or with 3 mM sodium fluoride first applied. The ordinates give force of contraction in milli Newton (mN, ordinates) over time in minutes (min, abscissae) in electrically stimulated mouse left atrium. The ratio of force at label ***b*** divided by force at label ***a*** marks the negative inotropic effect of carbachol. **G** Summarized data indicating the positive inotropic effect of 3 mM sodium fluoride over time. Data are given in mean values in percentage ± SEM. * and # first significant difference versus zero time point or Tyrode´s solution, respectively. Number of experiments *n* = 3. **H** Summarized data indicating the negative and positive inotropic effect of carbachol (1 µM) in presence or absence of sodium fluoride (3 mM) over time. Ordinate and abscissa give force of contraction in % of pre-drug value or time in minutes (min), respectively. * and # first significant difference versus zero time point (Ctr) or carbachol in presence of sodium fluoride, respectively. Number of experiments *n* = 3

Sodium fluoride (3 mM), like cantharidin, exerted a time-dependent positive inotropic effect in mouse left atrial preparations that was slow to develop (Fig. 6B). A time control experiment is displayed in Fig. 6A. Previously, we had performed concentration response curves of sodium fluoride in guinea pig papillary muscle and noted that 3 mM sodium fluoride was a concentration where phosphatase activity was already inhibited, force of contraction increased, but no toxic effects on contraction were apparent (Neumann et al. 1995a). The time dependency of the positive inotropic effect of sodium fluoride in mouse left atrium was quantified in Fig. 6G.

**Fig. 7:**
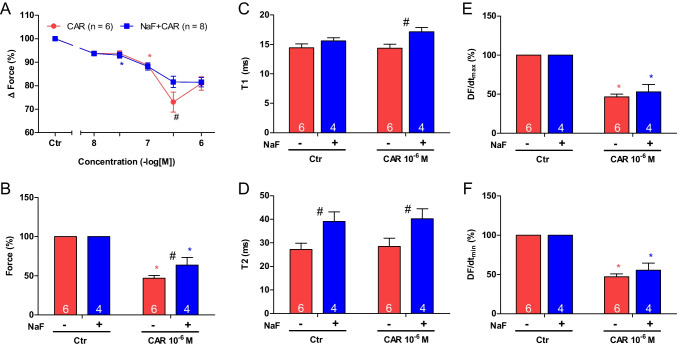
Sodium fluoride attenuates the effect of carbachol on contractile parameters in isolated electrically driven mouse left atrial preparations. **A** Line diagram of the negative inotropic effect of carbachol (10, 30, 100, 300 nM, 1 µM) alone or with first applied sodium fluoride (3 mM) in isolated electrically driven mouse left atrial preparations. Concentrations of carbachol applied before negative inotropic effect was reversed. Ordinate and abscissa give force of contraction in % of pre-drug value or applied concentration of carbachol, respectively. * and # first significant difference versus zero time point (Ctr) or carbachol in presence of sodium fluoride, respectively. Numbers (n) indicate number of experiments. **B** Bar diagram of the negative inotropic effect of carbachol (1 µM) alone or in the additional presence of sodium fluoride (3 mM) in isolated electrically driven mouse left atrial preparations. The effect of 3 mM sodium fluoride or time matched control values on force of contraction were set as 100 percent (left two columns). Negative inotropic effects of carbachol in the absence (-) or in the presence (+) of 3 mM sodium fluoride are plotted. * *p* < 0.05 vs. zero time point (Ctr). # *p* < 0.05 vs. without sodium fluoride. Ordinate: Force of contraction in %. Numbers in bars indicate number of experiments. **C** Bar diagram of the effect of carbachol (1 µM) alone or in the additional presence of sodium fluoride (3 mM) on time to peak tension in isolated electrically driven mouse left atrial preparations. The effect of 3 mM sodium fluoride or time matched control values for time to peak tension are shown as controls (Ctr) (left two columns). Next is displayed the effect of carbachol in the absence (-) or in the presence (+) of 3 mM sodium fluoride on time to peak tension. # *p* < 0.05 vs. without sodium fluoride. Ordinate: time to peak tension in milliseconds (ms). Numbers in bars indicate number of experiments. **D** Bar diagram of the effect of carbachol (1 µM) alone or in the additional presence of sodium fluoride (3 mM) on time of relaxation in isolated electrically driven mouse left atrial preparations. The effect of 3 mM sodium fluoride or time matched control values for time of relaxation are shown as control (Ctr) (left two columns). Next is displayed the effect of carbachol in the absence (-) or in the presence (+) of 3 mM sodium fluoride on time of relaxation. # *p* < 0.05 vs. without sodium fluoride. Ordinate: time of relaxation in milliseconds (ms). Numbers in bars indicate number of experiments. **E** Bar diagram of the effect of carbachol (1 µM) alone or in the additional presence of sodium fluoride (3 mM) on rate of tension development in isolated electrically driven mouse left atrial preparations. The effect of sodium fluoride or time matched control values on rate of tension development were set as 100 percent (left two columns). Effects of carbachol in the absence (-) or in the presence (+) of 3 mM sodium fluoride on rate of tension development are plotted. * *p* < 0.05 vs. zero time point (Ctr). Ordinate: rate of tension development in % . Numbers in bars indicate number of experiments. **F** Bar diagram of the effect of carbachol (1 µM) alone or in the additional presence of sodium fluoride (3 mM) on rate of tension relaxation in isolated electrically driven mouse left atrial preparations. The effect of sodium fluoride or time matched control values on rate of tension relaxation were set as 100 percent (left two columns). Effects of carbachol in the absence (-) or in the presence (+) of 3 mM sodium fluoride on rate of tension relaxation are plotted. * *p* < 0.05 vs. zero time point (Ctr). Ordinate: rate of tension relaxation in %. Numbers in bars indicate number of experiments

Like above for cantharidin, two different modes of application of carbachol were studied in order to discern in detail the contractile effects of carbachol. Firstly, carbachol (10 nM—1 µM) was cumulatively applied and the next higher concentration was added before the positive inotropic effect of carbachol could develop. Carbachol was studied alone (Fig. 6C) or in presence of firstly applied 3 mM sodium fluoride (Fig. 6D). It turned out that like cantharidin sodium fluoride can attenuate the negative inotropic effects of carbachol (Fig. 7A).

Secondly, we wanted to test the time course for the negative and positive inotropic effect of carbachol for 10 min. The data are summarized in Fig. 6H. It turned out that sodium fluoride can attenuate the negative inotropic effects of carbachol but not its positive inotropic effects (Fig. 6H).

In separate experiments, initially sodium fluoride was applied for 30 min as in Fig. 6B and then carbachol was applied as in Fig. 6E (1, 3, 10 µM). In the presence of sodium fluoride, the negative inotropic effects of carbachol at 1 µM are attenuated (Fig. 6F). These data are summarized in Fig. 7B. Likewise, under these conditions, the reductions of the rate of tension development and the time of relaxation by 1 µM carbachol were attenuated in the presence of sodium fluoride (Fig. 7D, E and Table 1), but time to peak tension was not altered by sodium fluoride (Fig. 7C). However, time to peak tension was prolonged by carbachol in the presence of sodium fluoride (Fig. 7C). The time of relaxation was prolonged by sodium fluoride alone (Fig. 7D), whereas additionally applied carbachol did not alter time of relaxation (Fig. 7D).

### Human right atrial preparations

**Fig. 8:**
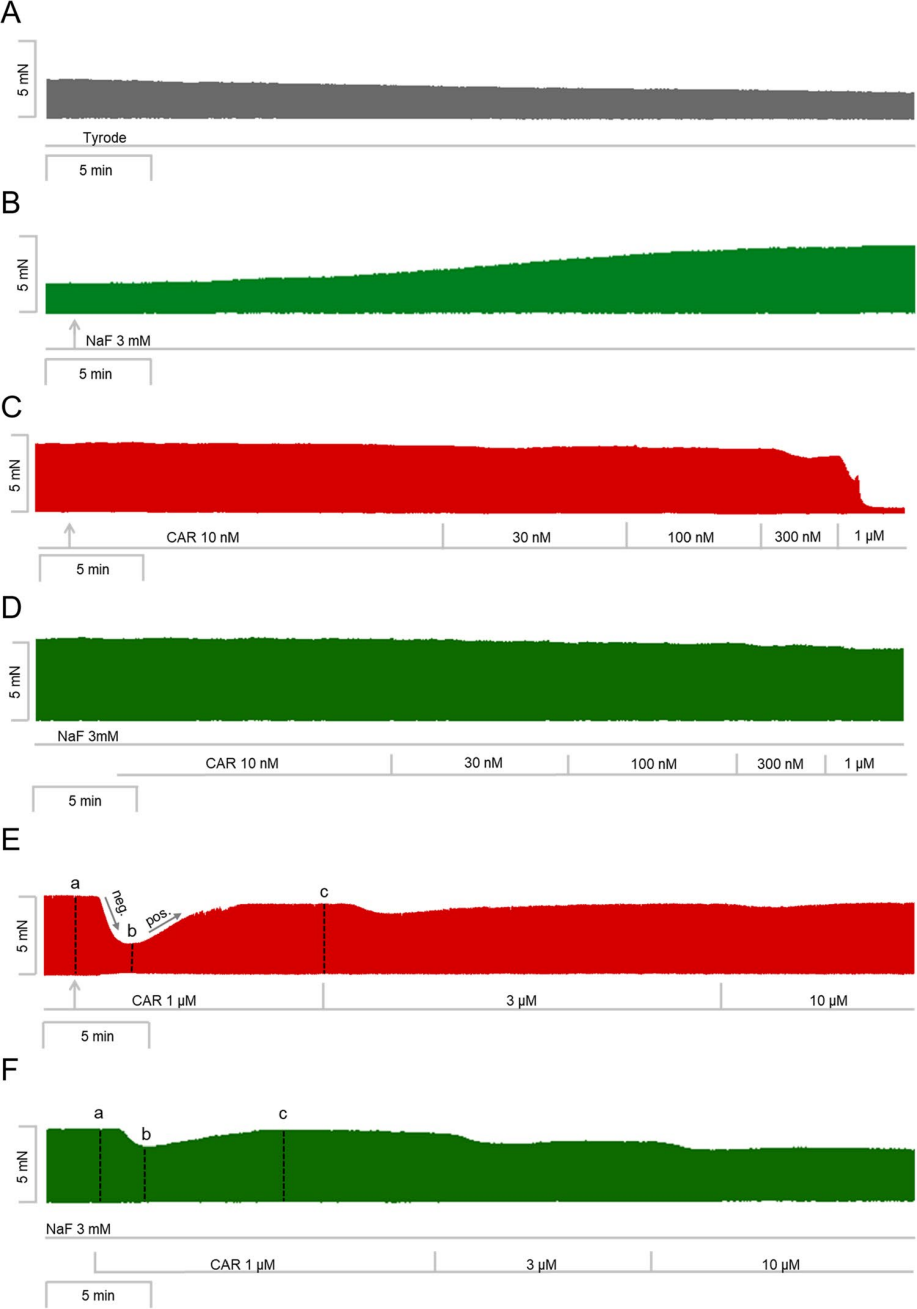

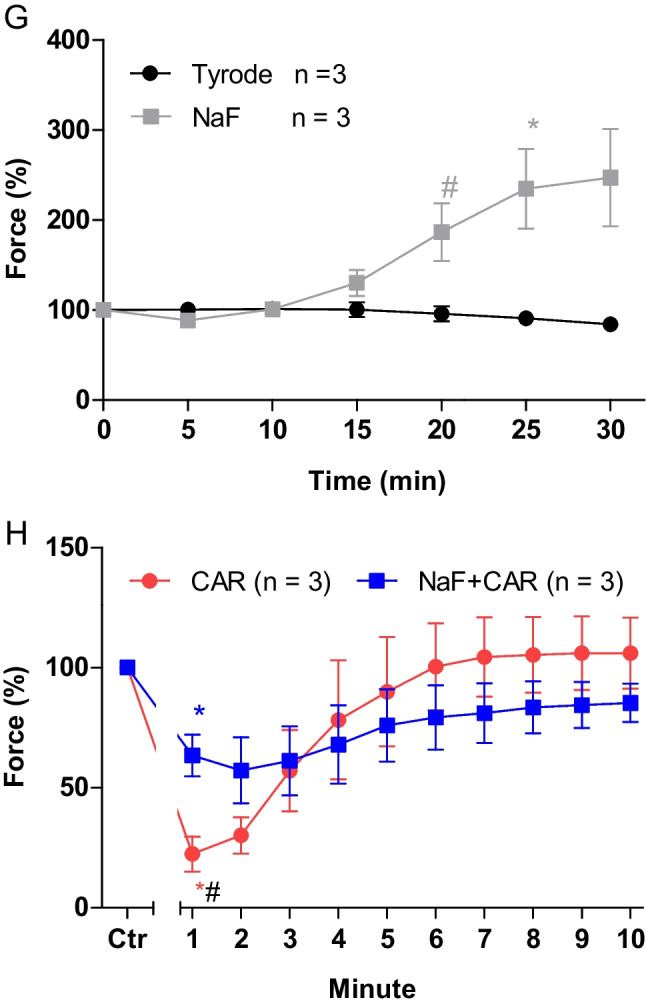
Sodium fluoride attenuates the negative inotropic effect of carbachol on force of contraction in human atrium**.** Original recording of the buffer, Tyrode´s solution, alone (**A**), in the presence of 3 mM sodium fluoride alone (**B**), after application of carbachol (10, 30, 100, 300 nM, 1 µM) alone (**C**) or with sodium fluoride (3 mM) first applied (**D**). Concentrations of carbachol applied before negative inotropic effect was reversed and after application of carbachol (1, 3, 10 µM) alone (**E**), or with first sodium fluoride and, when plateau was reached, thereafter carbachol (1, 3, 10 µM, **F**) applied. The ordinates give force of contraction in milli Newton (mN, ordinates) over time in minutes (min, abscissae) in electrically stimulated mouse left atrium. The ratio of force at label ***b*** divided by force at label ***a*** marks the negative inotropic effect of carbachol. **G** Summarized data indicating the positive inotropic effect of sodium fluoride over time. Data are given in mean values in percentage ± SEM. * and # first significant difference versus zero time point or Tyrode´s solution, respectively. Number of experiments *n* = 3. **H** Summarized data indicating the negative and positive inotropic effect of carbachol (1 µM) in presence or absence of sodium fluoride (3 mM) over time. Ordinate and abscissa give force of contraction in % of pre-drug value or time in minutes (min), respectively. * and # first significant difference versus zero time point (Ctr) or carbachol in presence of sodium fluoride, respectively. Number of experiments *n* = 3

Sodium fluoride (3 mM), like cantharidin exerted a time-dependent positive inotropic effect (Fig. 8B) compared to controls (Fig. 8A) in human atrial preparations. We decided to test 3 mM sodium fluoride also in the human atrium in order to facilitate comparison with our studies in mouse atrium, guinea pig papillary muscle, rat atrium and guinea pig atrium where we also studied 3 mM sodium fluoride (Neumann et al. 2005a, Neumann and Scholz 1998; Boknik et al. 2001). The time course of the positive inotropic effect of sodium fluoride was quantified in Fig. 8G.

**Fig. 9:**
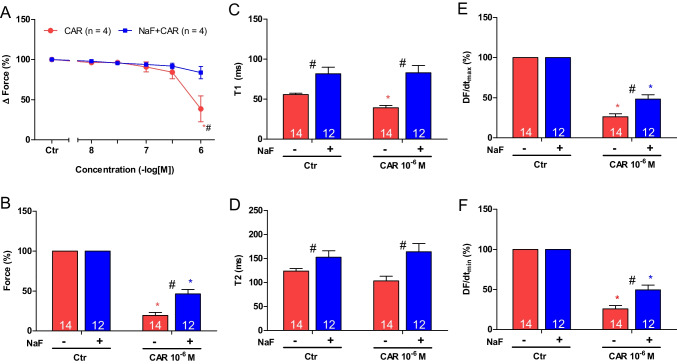
Sodium fluoride attenuates the effect of carbachol on contractile parameters in human atrium. **A** Line diagram of the negative inotropic effect of carbachol (10, 30, 100, 300 nM, 1 µM) alone or with first applied sodium fluoride (3 mM) in isolated electrically driven human right atrial preparations. Concentrations of carbachol applied before negative inotropic effect was reversed. Ordinate and abscissa give force of contraction in % of pre-drug value or applied concentration of carbachol, respectively. * and # first significant difference versus zero time point (Ctr) or carbachol in presence of sodium fluoride, respectively. Numbers (n) indicate number of experiments. **B** Bar diagram of the negative inotropic effect of carbachol (1 µM) alone or in the additional presence of sodium fluoride (3 mM) in isolated electrically driven human right atrial preparations. The effect of 3 mM sodium fluoride or time matched control values on force of contraction were set as 100 percent (left two columns). Negative inotropic effects of carbachol in the absence (-) or in the presence (+) of 3 mM sodium fluoride are plotted. * *p* < 0.05 vs. zero time point (Ctr). # *p* < 0.05 vs. without sodium fluoride. Ordinate: Force of contraction in %. Numbers in bars indicate number of experiments. **C** Bar diagram of the effect of carbachol (1 µM) alone or in the additional presence of sodium fluoride (3 mM) on time to peak tension in isolated electrically driven human right atrial preparations. The effect of 3 mM sodium fluoride or time matched control values for time to peak tension are shown as control (Ctr) (left two columns). Next is displayed the effect of carbachol in the absence (-) or in the presence (+) of 3 mM sodium fluoride on time to peak tension. * *p* < 0.05 vs. zero time point (Ctr). # *p* < 0.05 vs. without sodium fluoride. Ordinate: time to peak tension in milliseconds (ms). Numbers in bars indicate number of experiments. **D** Bar diagram of the effect of carbachol (1 µM) alone or in the additional presence of sodium fluoride (3 mM) on time of relaxation in isolated electrically driven human right atrial preparations. The effect of 3 mM sodium fluoride or time matched control values for time of relaxation are shown as control (Ctr) (left two columns). Next is displayed the effect of carbachol in the absence (-) or in the presence (+) of 3 mM sodium fluoride on time of relaxation. # *p* < 0.05 vs. without sodium fluoride. Ordinate: time of relaxation in milliseconds (ms). Numbers in bars indicate number of experiments. **E** Bar diagram of the effect of carbachol (1 µM) alone or in the additional presence of sodium fluoride (3 mM) on rate of tension development in isolated electrically driven human right atrial preparations. The effect of sodium fluoride or time matched control values for rate of tension development were set as 100 percent (left two columns). Effects of carbachol in the absence (-) or in the presence (+) of 3 mM sodium fluoride on rate of tension development are plotted. * *p* < 0.05 vs. zero time point (Ctr). # *p* < 0.05 vs. without sodium fluoride. Ordinate: rate of tension development in %. Numbers in bars indicate number of experiments. **F** Bar diagram of the effect of carbachol (1 µM) alone or in the additional presence of sodium fluoride (3 mM) on rate of tension relaxation in isolated electrically driven human right atrial preparations. The effect of sodium fluoride or time matched control values for rate of tension relaxation were set as 100 percent (left two columns). Effects of carbachol in absence (-) or presence (+) of 3 mM sodium fluoride on rate of tension relaxation are plotted. * *p* < 0.05 vs. zero time point (Ctr). # *p* < 0.05 vs. without sodium fluoride. Ordinate: rate of tension relaxation in %. Numbers (n) indicate number of experiments

Again, two different modes of application of carbachol were studied. In the first set of experiments, carbachol (10 nM—1 µM) was cumulatively applied, and the next higher concentration was added before the positive inotropic effect of carbachol could develop. Carbachol alone (Fig. 8C) or carbachol in presence of 3 mM sodium fluoride (Fig. 8D) were studied. The data are summarized in Fig. 9A. It turned out that sodium fluoride like cantharidin can attenuate the negative inotropic effects of carbachol also in human atria (Fig. 9A).

In the following second set of experiments, we examined the time course of the negative and positive inotropic effect of carbachol for 10 min. Here, instead of performing a concentration response curve as in Fig. 8C, only 1 µM carbachol was added and the effect was quantified each minute for ten minutes. The data are summarized in Fig. 8H. Sodium fluoride attenuated the negative inotropic effects of 1 µM carbachol but only transiently (Fig. 8H).

In the next experiments, the preparations were incubated initially with sodium fluoride for 30 min as in Fig. 8B and then carbachol was cumulatively applied as in Fig. 8E (1, 3, 10 µM). In the presence of sodium fluoride, the negative inotropic effects of carbachol at 1 µM were attenuated as shown in Fig. 8F and data are summarized in Fig. 9B. Likewise, under these conditions, the reductions of the rate of tension development and time of relaxation by 1 µM carbachol were attenuated in the presence of sodium fluoride (Fig. 9D, E and Table 1). Time to peak tension was prolonged by sodium fluoride (Fig. 9C) whereas time to peak tension was not altered by carbachol in the presence of sodium fluoride (Fig. 9C). Sodium fluoride alone was able to prolong time of relaxation (Fig. 9D), but additionally applied carbachol did not alter time of relaxation (Fig. 9D).

## Discussion

The main new finding in this report: cantharidin and sodium fluoride attenuate the negative inotropic effect of carbachol in the human atrium.

Of note, cantharidin is more potent in mouse than in human atrium to increase force of contraction like sodium fluoride, as reported by us before (Schwarz et al. 2023b). The contractile effects of sodium fluoride are also species- and region-dependent. For instance, we noted a positive inotropic effect of sodium fluoride in guinea pig papillary muscle (Neumann et al. 1995a; Neumann and Scholz 1998). In contrast, sodium fluoride decreased force of contraction in guinea pig atrium and rat atrium until the preparations failed to contract (Neumann and Scholz 1998). Hence, novel in the present work is that sodium fluoride increased force of contraction in mouse atrium and more importantly in human atrium. Thus, at least in this regard, mouse and human heart behave similarly.

We had shown before that sodium fluoride inhibited the enzymatic activity of serine/threonine phosphatases, namely PP1 and PP2A, from guinea pig hearts (Neumann et al. 1995a). In guinea pig and human ventricular preparations, in contrast to atrial preparations, carbachol alone does not decrease force of contraction (Dhein et al. 2001). However, when force was pre-stimulated by isoprenaline (a β-adrenoceptor agonist) or 3-isobutyl-1-methylxanthine (an unselective phosphodiesterase inhibitor), carbachol induced a time- and concentration-dependent negative inotropic effect in guinea pig papillary muscle that was blocked by 3 mM sodium fluoride (Neumann et al. 1995a).

Hence, with the present report, we have extended our studies to atrial preparations. In mouse atrium and human atrium, the direct negative inotropic effects of carbachol were attenuated by previously applied sodium fluoride. This is not proof that the action of sodium fluoride is solely due to inhibition of phosphatases. Admittedly, sodium fluoride has a number of other effects in the biochemistry of the heart (discussed in Neumann and Scholz 1998). However, taking into account that cantharidin acted similarly, it is a viable hypothesis that under our experimental conditions, sodium fluoride used phosphatase inhibition to antagonize the effects of carbachol.

We have measured in this report the time to peak tension and the rate of tension development because it may be a valid functional read out for the activity of phosphatases in the heart. The time to peak tension is shortened after β-adrenergic stimulation of the cardiomyocyte. This is in part mediated by activation of the cAMP dependent protein kinase, and in part by activation of a calcium/calmodulin dependent kinase. The kinases phosphorylate and thereby facilitate the opening of the ryanodine receptor (Fig. 1): more calcium ions in a given time leave the sarcoplasmic reticulum and thus calcium ion concentration increases faster near the myofilaments and contraction starts earlier in systole. Now, if we assume that cantharidin and sodium fluoride inhibit the phosphatases in the vicinity of the ryanodine receptor located in the junctional sarcoplasmic reticulum, the phosphorylation state of the ryanodine receptor might increase or at least its dephosphorylation might occur slower. In consequence, phosphatase inhibition via this pathway may shorten time to peak tension.

Carbachol if it activates phosphatases might have the opposite effect. However, carbachol can also open potassium channels and close LTCC, conceivably without phosphatases as intermediators.

A similar mechanism might explain alterations in mechanical relaxation. Relaxation of the heart occurs faster if in a given time more calcium ions are pumped from the cytosol into the sarcoplasmic reticulum, where it is stored mainly by binding to calsequestrin (Fig. 1). The pump involved is SERCA. SERCA activity is inhibited by dephosphorylated phospholamban. As delineated above, β-adrenergic stimulation of the cardiomyocyte will activate kinases that phosphorylate phospholamban (Fig. 1). Once phospholamban is phosphorylated it may dissociate from SERCA. In any case, SERCA activity increases when phospholamban is phosphorylated and calcium ions more quickly enter the sarcoplasmic reticulum. This leads to faster tension relaxation in diastole. Now, as discussed above, if phosphatases that are in the vicinity of phospholamban in the free sarcoplasmic reticulum are inhibited, increased phosphorylation of phospholamban occurs. This is not hypothetical. We have reported years ago that phosphatase inhibitors like okadaic acid, calyculin A and cantharidin increase the phosphorylation state of phospholamban in the guinea pig ventricle (in perfused heart, papillary muscles and isolated ventricular cardiomyocytes, Neumann et al. 1993; Neumann et al. 1994a, b; Neumann et al. 1995a, b; Boknik et al. 2001). Recently, we could show that cantharidin increased the phosphorylation state of phospholamban in contracting human right atrial muscle strips (Schwarz et al. 2023a, b). An increase in phospholamban phosphorylation leads to faster relaxation.

That cantharidin leads to faster relaxation, we have shown before in guinea pig and human atrial preparations (Schwarz et al. 2023b). However, if one assumes that carbachol activates phosphatases in the vicinity of phospholamban, one would predict carbachol should prolong relaxation. Again, carbachol also opens potassium channels and closes LTCC in the heart. This alone would shorten the duration of the action potential and thus, in a way independent of phospholamban phosphorylation, would shorten the time of mechanical contraction and thus would reduce the time available for contraction.

An open question in the field, despite our and others´ past efforts, is the question how cardiac M_2_-receptors exactly couple to phosphatases. An approach for further work is the use of knockout mice for proteins suspected to anchor phosphatases to the M_2_-receptor. But this is obviously the subject of ongoing research.

We have shown previously on mRNA and protein levels that the catalytic subunits of PP1 and PP2A are present in the human atrium and in the human ventricle (Lüss et al. 2000). Hence, the targets of cantharidin are present in the human atrium.

The cardiac action of carbachol is different between species and between cardiac regions namely atrium and ventricle. If we look firstly to the atrium, functionally negative inotropic effects of carbachol are well established in human atrium and mouse atrium (e.g. Boknik et al. 2009; Du et al. 1994; Neumann et al. 2003). Moreover, in human and mouse atrium, a secondary positive inotropic effect is always seen in our lab and by others (Boknik et al. 2009; Du et al. 1994; Neumann et al. 2003). The negative inotropic effect of carbachol vanishes in atrium from M_2_-muscarinic receptor knockout mice and thus is M_2_-muscarinic receptor mediated (review: Dhein et al. 2001). The positive inotropic effect of carbachol is missing in M_3_-muscarinic receptor knockout mice and thus in all likelihood appears to be M_3_-muscarinic receptor-mediated (Dhein et al. 2001). Similar findings are seen in human atrial samples, where studies with receptor specific antagonists come to the same conclusions (Dhein et al. 2001). However, also the presence of M_1_-muscarinic receptors in the human atrium has been described (Heijman et al. 2018). Possibly, this species difference may be responsible that in mice, cantharidin did not suppress the positive inotropic effect of carbachol, whereas in humans, this was the case. Moreover, the M_3_-muscarinic receptor, responsible for the positive inotropic effect, is postulated to be located to the endocardial endothelium (Hara et al. 2009) and, therefore, the positive inotropic effect of carbachol is assumed to be indirect and, hence, differentially regulated by protein phosphatases in mice and humans. Thus, on the one hand, our negative inotropic effects of carbachol in mouse and human atrium are probably M_2_-muscarinic receptor-mediated. On the other hand, we have repeatedly shown that the negative inotropic effect of carbachol in the presence of isoprenaline in the guinea pig ventricle involves phosphorylation of the phosphatase inhibitor 1 and subsequent inhibition of PP1 (Gupta et al. 1993, 1994, 1998; Neumann et al. 1994a, b). Others presented evidence that in the ventricle, carbachol can also activate PP2A (Chu et al. 2003). Hence, there is precedence that carbachol can activate PP1 and PP2A in the mammalian heart. We postulate now that the same occurs in the human atrium. Carbachol tries to activate PP1 and PP2A in the human atrium, but this is impaired by cantharidin and sodium fluoride. We suggest that the remaining negative inotropic effect of carbachol in the permanent presence of cantharidin is due to activation of potassium ion or inhibition of calcium ion channels in the atrium, in a phosphorylation independent fashion (Dhein et al. 2001).

Limitations of the study: We cannot offer a biochemical chain of events from the receptor to the phosphatase in the human atrium. We do not know whether cantharidin and sodium fluoride act in a similar way in the human ventricle as in the human atrium, because we have currently no access to that tissue. Moreover, the mechanisms of phosphatase(s) activation by stimulation of muscarinic receptors could well be different in mouse atrium and human atrium. To name a few differences between these species, some G-protein coupled receptors are lacking in mouse atrium that are effective in human atrium (5-HT_4_-serotonin-receptors, H_2_-histamine receptors: Gergs et al. 2010, 2019). Hence, while serotonin increases Ca^2+^ transients in human hearts, such effects are lacking in wild type mice (Gergs et al. 2010). Another example would be that expression of Ca^2+^ regulatory proteins in the heart is different in mice and men (Lüss et al. 2000). A further limitation of the study resides in the fact that we cannot readily explain why sodium fluoride prolongs time parameters while it increases force of contraction. One would have expected, as delineated above, a shortening of the duration of contraction. Hence, other effects of sodium fluoride for instance, detrimental effects on mitochondria are known and might play a role.

However, we and others have generated mice with overexpression, knock out or knock down of cardiac phosphatases (review: Neumann et al. 2021). Based on our initial data presented here for the mouse, it may be useful to study cantharidin in these transgenic mice to test our assumptions. We are planning such studies in our transgenic models.

In conclusion, we can now answer the hypotheses put forward in the Introduction in the following way. Cantharidin and sodium fluoride attenuate the negative inotropic effects of carbachol in mouse and human atrium. We speculate that cantharidin and sodium fluoride inhibit cardiac phosphatases that carbachol would stimulate.

## Data Availability

The data of this study are available from the corresponding author upon reasonable request.
